# Optic Disc Hemorrhage Is Related to Various Hemodynamic Findings by Disc Angiography

**DOI:** 10.1371/journal.pone.0120000

**Published:** 2015-04-16

**Authors:** Hae Young Lopilly Park, Hyun Jin Jeong, Yoon Hee Kim, Chan Kee Park

**Affiliations:** Department of Ophthalmology and Visual Science, Seoul St. Mary's Hospital, College of Medicine, The Catholic University of Korea, Seoul, Korea; Bascom Palmer Eye Institute, University of Miami School of Medicine, UNITED STATES

## Abstract

**Background:**

To investigate the hemodynamic characteristics of glaucoma eyes with disc hemorrhage (DH) by disc fluorescein angiography, and its relationship with glaucomatous changes of the optic disc and surrounding retinal nerve fiber layer (RNFL).

**Methods:**

This study included 35 glaucoma eyes with DH who were followed up at least 5 years and had DH at presentation. Eyes were classified as eyes with DH at the border of localized RNFL defects and eyes with DH not related to localized RNFL defects. Prevalence of DH and location of the proximal border were recorded from disc photographs. Fluorescein angiography was performed 3 months after detecting the DH. Arm-retina time, arteriovenous transit time, disc filling time, choroidal filling time, and venous filling time were measured as retinal circulation parameters. The presence of disc filling defects and disc leaks were evaluated.

**Results:**

There were 19 (54.3%) eyes with DH accompanying localized RNFL defects. The arm-retina time was prolonged in eyes with DH not related to RNFL defects (*P* = 0.044) and the arteriovenous transit time was prolonged in eyes with DH accompanying RNFL defects (*P* = 0.029). Among eyes with DH accompanying RNFL defects, 11 (57.9%) had vessel filling defects or delayed filling indicating blood flow stasis at the cup margin proximal to where DH occurred. Eyes with DH not related to RNFL defects did not show vessel filling defects or delayed filling.

**Conclusions and Relevance:**

Eyes with DH related to RNFL defects showed prolonged arteriovenous transit time and had frequent vessel filling defects or delayed filling indicating blood flow stasis and thrombus formation at the site DH occurred. These findings suggest that vascular and hemodynamic changes due to glaucomatous structural changes cause DH in relation to localized RNFL defects.

## Introduction

Disc hemorrhage (DH) is a prominent feature of glaucoma.[1, 2] The Ocular Hypertension Treatment Study demonstrated that DH is a significant risk factor for the development of glaucoma.[3] Eyes with DH were six times more likely to develop glaucoma than eyes without a DH. DH was significantly associated with glaucoma progression in the Early Manifest Glaucoma Trial and Collaborative Normal-Tension Glaucoma Study.[4, 5] Eyes with DH progressed more rapidly than eyes without a DH. DH has shown to be related to clear clinical importance in glaucoma.

Typically, DH related to glaucoma is located in the prelaminar region of the optic nerve head (ONH) and the surrounding superficial retinal nerve fiber layer (RNFL). DH has been proposed that structural changes at the level of the lamina cribrosa cause the mechanical rupture of small blood vessels, leading to DH.[6] DH has been associated with notching, progressive changes in the optic disc rim, localized RNFL defects, and enlargement of localized RNFL defects.[7–10] Normal-tension glaucoma (NTG) eyes with DH have a thinner lamina cribrosa.[11] These findings suggest that structural changes at the lamina cribrosa, optic disc, or RNFL may contribute to mechanical rupture of vessels, leading to DH. However, lowering IOP does not increase the frequency of DH.[4, 12] Additionally, DH is more frequent in NTG than primary-open angle glaucoma (POAG) with high IOP.[2] NTG is considered to be strongly affected by non-IOP factors, particularly vascular factors, more than POAG.[13–17] DH has also been described in patients with primary vascular dysregulation, a syndrome of decreased ocular perfusion and systemic blood pressure.[18] In these cases, DH may develop due to vascular effects, including microinfarction within the ONH and localized vascular insufficiency at the ONH.[6, 9]

DHs are transient phenomena, usually lasting 6–12 weeks.[19] However, the underlying pathogenesis may be important to understand the clinical significance of DH. This is why studies focus on revealing the underlying pathogenesis of DH in eyes with glaucoma. Also, the clinical significance of recurrent DH is not well understood. There are controversies whether that recurrent DH adds negative prognostic factor to single DH or not.[20–22] However, DH tends to occur within 2 clock hours of an area of neuroretinal rim notching or RNFL defect, and recur within 2 clock hours of the initial DH. This may reflect the relationship between DH and glaucomatous structural changes of the optic disc or RNFL.[22]

In this study, we investigated the blood flow at the ONH observed by disc fluorescein angiography and its relationship with glaucomatous change of the optic disc and surrounding RNFL in eyes with DH. We aimed to determine the relationship between glaucomatous structural change of the ONH and the accompanying hemodynamic changes and their contribution to the development of DH.

## Methods

### Subjects

We enrolled open-angle glaucoma patients with DH who were seen by a glaucoma specialist (CKP) from January 2012 to August 2012 at the glaucoma clinic of the Seoul St. Mary’s Hospital, Seoul, Korea. Patients who were followed up at least 5 years in the past were selected for the study. The medical records of these patients with DH were thoroughly reviewed and all disc photographs of each patient from the initial presentation to the last follow-up were evaluated. Patients received disc fluorescein angiography 3 months after enrollment. For the initial work-up, each patient received a complete ophthalmologic examination, including a review of the medical history, measurement of best-corrected visual acuity, refraction, slit-lamp biomicroscopy, gonioscopy, Goldmann applanation tonometry, central corneal thickness using ultrasound pachymetry (Tomey Corporation, Nagoya, Japan), axial length using ocular biometry (IOL Master; Carl Zeiss Meditec), dilated stereoscopic examination of the optic disc, disc and red-free fundus photography (Canon, Tokyo, Japan), and Humphrey visual field (VF) examination using the Swedish interactive threshold algorithm Standard 24–2 test (Carl Zeiss Meditec, Dublin, CA).

For glaucoma diagnosis, patients had to fulfill the following criteria: glaucomatous optic disc appearance (such as diffuse or localized rim thinning, a notch in the rim, or a vertical cup-to-disc ratio higher than that of the other eye by more than 0.2) and glaucomatous VF loss (defined as a pattern standard deviation [P < 0.05] or glaucoma hemifield test results [P < 0.01] outside the normal limits in a consistent pattern on two qualifying VFs), both confirmed and agreed on by two glaucoma specialists (HYP, CKP); and an open angle on gonioscopic examination.

All patients had to meet the following additional inclusion criteria to be entered into the study: a best-corrected visual acuity ≥20/40, a spherical refraction within ±6.0 diopters, a cylinder correction within ±3.0 diopters, consistently reliable VFs (defined as false negative < 15%, false positive < 15%, and fixation losses < 20%), and mean deviation (MD) better than -12.00 dB. Patients were excluded on the basis of any of the following criteria: history of diabetes mellitus or systemic hypertension, taking drugs for treatment of systemic hypertension or heart disease, such as calcium channel blockers, angiotension-converting enzyme inhibitors, β-adrenergic blockers, and α-1 adrenergic blockers, a history of any retinal disease, including diabetic or hypertensive retinopathy, a history of eye trauma or surgery with the exception of uncomplicated cataract surgery, other optic nerve disease except for glaucoma, a history of systemic or neurologic diseases that might affect the VF. If both eyes of a glaucoma patient had disc hemorrhage and met the inclusion and exclusion criteria, one eye was randomly chosen for the study.

The Institutional Review Board from the Seoul St. Mary’s Hospital approved the study and the study adhered to the principles of the Declaration of Helsinki. Written informed consent was obtained from each participant after the agreement to participate in the study.

### Disc and red-free photographs

Two glaucoma specialists (HJJ and YHJ) evaluated the serial disc and red-free photographs during the total follow-up period in each patient. During follow-up period, patients have taken serial disc and red-free fundus photographs, regularly with intervals of 6–12 months. Interval of photograph was 6 months for the first two years and 12 months thereafter. Observers were blinded to the patient’s clinical information and test results.

A DH was defined as an isolated flame-shape or splinter-like hemorrhage on the optic disc or peripapillary area extending to the border of the optic disc. The observers described the clock-hour position of the DH in terms of the proximal locations. Proximal location means the end of the hemorrhage closest to the disc center. Eyes with DH were divided into four types according to the proximal location (within cup base, cup margin, disc margin, and within peripapillary atrophy). The number of DHs that occurred during 5-year follow-up was recorded (DH at enrollment was counted in the number). DH occurring at the borders of a localized RNFL defect was classified as ‘DH accompanying RNFL defect.’ Discrepancies in evaluating the presence of DH, proximal location of DH, and DH accompanying RNFL defect between the two observers were resolved by consensus.

### Optic disc fluorescein angiography

At 3 months after the detected DH, patients were scheduled to undergo fluorescein angiography. Fluorescein angiography of the ONH was performed with scanning laser ophthalmoscopy (Heidelberg Retina Angiograph, Heidelberg Engineering, Heidelberg, Germany). All subjects gave informed consent concerning the side effects of fluorescein angiography. To start the angiography, 10% sodium fluorescein dye/70kg body weight was injected into an antecubital vein. The video timer was started as fluorescein dye was injected. Records were taken at 1-second intervals from the first entrance to the retina to the late venous phase. This lasted for 30 seconds, and late-phase photos were taken after an interval of 2 to 3 minutes. Retinal photographs were obtained using a Topcon IMAGEnet 2000 TRC-50 retinal camera (Topcon, Capelle aan den IJssel, The Netherlands) with the exclusion of red light.

Arm-retina time, arteriovenous transit time, disc filling time, choroidal filling time, and venous filling time were measured as retinal circulation parameters. The normative values of each parameter are previously reported.[23] The video fluorescein angiograms were enlarged and examined visually by 2 researchers. Arm-retina time is described as the time from the administration of an opaque substance into the antecubital vein until it becomes visible in the retinal arteries. Arteriovenous transit time is the shortest time of retinal microcirculation and is the same as the early arteriovenous phase; it is described as the time period between the entrance of the opaque substance into the edge of the optic disc or into the retinal artery from a distance of 2 optic discs and the appearance of the opaque substance in the vein at the same point. In the present study, measurements were obtained from the temporal region from an average distance of 2 optic discs. Disc filling time is described as the time from when the fluorescein first appears in the disc until the disc is totally filled. Choroidal filling time is described as the time from when the fluorescein first appears in the choroid until the choroid is maximally filled. Venous filling time is described as the time from when the opaque substance first appears in the vein until the complete filling of vein (without laminar flow).

Images of the early phase (<3 minutes) visualized the superficial capillaries of the ONH. Disc leak was defined as when the gray level of the fluorescence of the optic disc was greater than the fluorescence level of the peripapillary retina at the late phase. Disc filling defects were defined as the presence of areas of persisting hypofluorescence during the whole angiogram, which corresponds to areas of superficial capillary loss.

### Statistical Analysis

The Mann-Whitney U test and chi-square test or Fisher’s exact test for independent samples were used to assess the differences between groups. A probability value of *P* < 0.05 was considered statistically significant. SPSS for Windows (ver. 12.0.0, SPSS Inc, Chicago, IL) was used for the statistically analyses.

## Results

A total of 36 eyes of 36 glaucoma patients with DH who met the inclusion and exclusion criteria underwent fluorescein angiography of the ONH. Retinal photographs from 1 patient had poor image quality and were excluded from the analysis. The mean age of the remaining 35 patients was 54.69 ± 1.24 years. The mean deviation of the visual field was -3.55 ± 4.33 dB in these patients. The mean number of DHs during follow-up was 1.83 ± 1.15 (range, 1–4), and the location of DHs was mainly the inferotemporal position (6.67 ± 1.38 clock-hour). The demographic details of the patients are listed in Table 1.

**Table 1:** Subject Characteristics.

Mean follow-up periods, years	6.25 ± 0.75
Age, years	54.69 ± 1.24
Gender (Male: Female)	16:19
Intraocular pressure, mmHg	15.82 ± 3.04
Axial length, mm	24.36 ± 1.40
Spherical equivalent, diopters	-2.16 ± 2.91
Central corneal thickness, μm	534.12 ± 3.30
Mean deviation, decibel	-3.55 ± 4.33
Pattern standard deviation, decibel	4.14 ± 3.72
Number of disc hemorrhage, n	1.83 ± 1.15
Location of disc hemorrhage, clock-hour	6.67 ± 1.38

Data are presented as mean ± standard deviation.

The mean arm-retina time was 20.97 ± 4.61 seconds, arteriovenous transit time was 2.33 ± 1.48 seconds, disc filling time was 9.93 ± 4.79 seconds, choroidal filling time was 11.96 ± 6.60 seconds, and venous filling time was 13.80 ± 4.91 seconds. The normal reference values are 10–14 seconds for arm-retina time, 1.2–2.1 seconds for arteriovenous transit time, 4–6 seconds for disc filling time, 5–7 seconds for choroidal filling time, and 10–12 seconds for venous filling time.[23] All parameters were prolonged compared to the normal values. There were 12 eyes (34.3%) with disc leaks and 12 eyes (34.3%) with disc filling defects, which are previously reported to be observed in less than 20% in normal eyes.[24–26]

There were 19 eyes (54.3%) with DH located at the borders of the localized RNFL defect (Table 2). The number of DHs per year was greater in eyes with the DH accompanying a RNFL defect (0.35 ± 0.10) compared to eyes with the DH not related to a RNFL defect (0.22 ± 0.07, *P* = 0.034). The proximal location of DH was significantly different between two groups (*P* = 0.003). In both groups, the proximal location of DH was most frequently the cup margin (78.9% in eyes with DH accompanying a RNFL defect; 43.8% in eyes with DH not related to a RNFL defect). However, there was no eye with the proximal location of DH within the rim area or cup base in eyes with DH accompanying a RNFL defect, which was frequent in eyes with DHs not related to RNFL defects (43.8%).

**Table 2:** Comparison of baseline characteristics and features of disc hemorrhage (DH) between glaucoma eyes with DH occurring at the border of the localized retinal nerve fiber layer (RNFL) defect and DH occurring not related to RNFL defect.

	Accompanying RNFL defect	Not related to RNFL defect	*P* Value
	(n = 19)	(n = 16)	
Mean follow-up periods, years	6.14 ± 0.65	6.29 ± 0.83	0.820*
Age, years	55.05 ± 12.94	54.25 ± 12.05	0.663*
Gender (Male: Female)	9:10	7:9	0.551^†^
Intraocular pressure, mmHg			
Mean	15.42 ± 2.89	16.31 ± 4.37	0.135*
Maximum	17.38 ± 2.46	17.24 ± 2.63	0.372*
Fluctuation	2.42 ± 1.27	2.35 ± 1.32	0.240*
Axial length, mm	24.32 ± 1.43	24.41 ± 1.44	0.380*
Spherical equivalent, diopters	-2.38 ± 3.01	-1.89 ± 2.89	0.751*
Central corneal thickness, μm	537.62 ± 27.24	530.33 ± 39.24	0.381*
Mean deviation, decibel	-3.58 ± 2.96	-3.44 ± 2.51	0.404*
Pattern standard deviation, decibel	4.11 ± 3.74	4.20 ± 3.38	0.650*
Systolic blood pressure, mmHg	127.54 ± 12.94	126.38 ± 13.05	0.523*
Diastolic blood pressure, mmHg	74.96 ± 12.30	75.23 ± 12.28	0.646*
MOPP, mmHg	52.38 ± 3.93	51.74 ± 4.26	0.722*
Use of topical β-blocker, n (%)	7 (36.8%)	6 (37.5%)	0.621^†^
Number of DH per year, n	0.35 ± 0.10	0.22 ± 0.07	0.034*
Location of DH, clock-hour	7.14 ± 1.46	6.35 ± 1.57	0.420*
Proximal location of DH			0.003^‡^
Cup margin, n (%)	15 (78.9%)	7 (43.8%)	
Disc margin, n (%)	3 (15.8%)	1 (6.2%)	
Within peripapillary atrophy, n (%)	1 (5.3%)	1 (6.2%)	
Within cup base, n (%)	0	7 (43.8%)	

Data are presented as mean ± standard deviation.

RNFL = retinal nerve fiber layer; DH = disc hemorrhage.

* Mann-Whitney U test.

^†^ Chi-square test.

^‡^ Fisher’s exact test.

**Fig 1:**
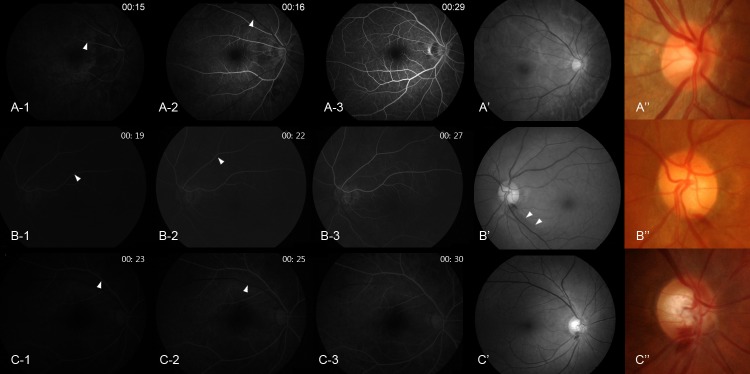
Fluorescein angiography of normal control eye (A) and glaucoma eyes with disc hemorrhage (DH) (B and C). The angiography of normal control eye was from a patient who underwent this examination due to unknown blurred vision (this case is not included in the analysis of the present study, however, is added in this figure to help comparison with glaucoma eyes). Arteriovenous transit time is the time between the entrance of the opaque substance into the retinal artery (arrowhead in A-1, B-1, C-1) and entrance into the vein (arrowhead in A-2, B-2, C-2) from a distance of 2 optic discs. Glaucomatous eye with DH occurring at the border of localized retinal nerve fiber layer (RNFL) defect (B’ and B”; localized RNFL defect, arrowhead in B’) shows prolonged arteriovenous transit time (time interval between B-1, when fluorescein dye is introduced into the artery, and B-2, when fluorescein dye enters the vein from a distance of 2 optic discs) (delayed by 3 seconds). Glaucomatous eye with DH occurring at the 6 o’clock position not related to a localized RNFL defect (C’ and C”) shows prolonged arm-retina time, which is the time from the administration of fluorescein dye to the antecubital vein until it becomes visible in the retinal arteries (C-1, 23 seconds).

**Fig 2:**
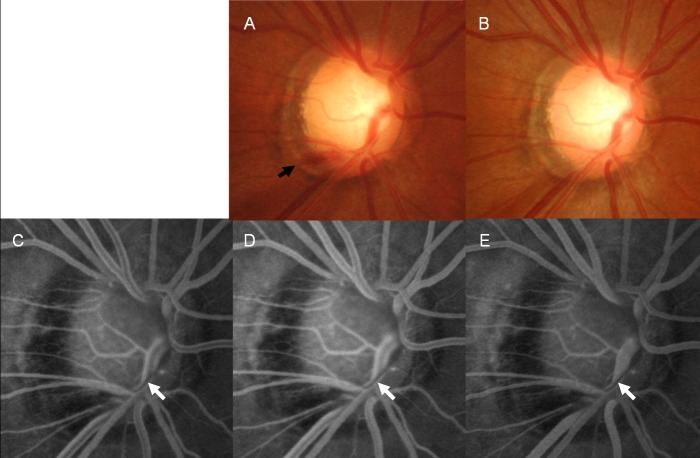
A 47-year-old female with normal-tension glaucoma. Disc hemorrhage (DH, black arrow) was present at the border of a localized retinal nerve fiber layer defect in her right eye (A). Fluorescein angiography was performed 3 months after the disappearance of the DH in the disc photograph (B). A vessel filling defect (white arrow) was found at the mid-arteriovenous phase (C), early late phase (D), and late phase of fluorescein angiography (E). The vessel filling defect was proximal to the location of the DH. This vein is reflected at the cup margin, and the proximal location of DH is at the cup margin.

**Fig 3:**
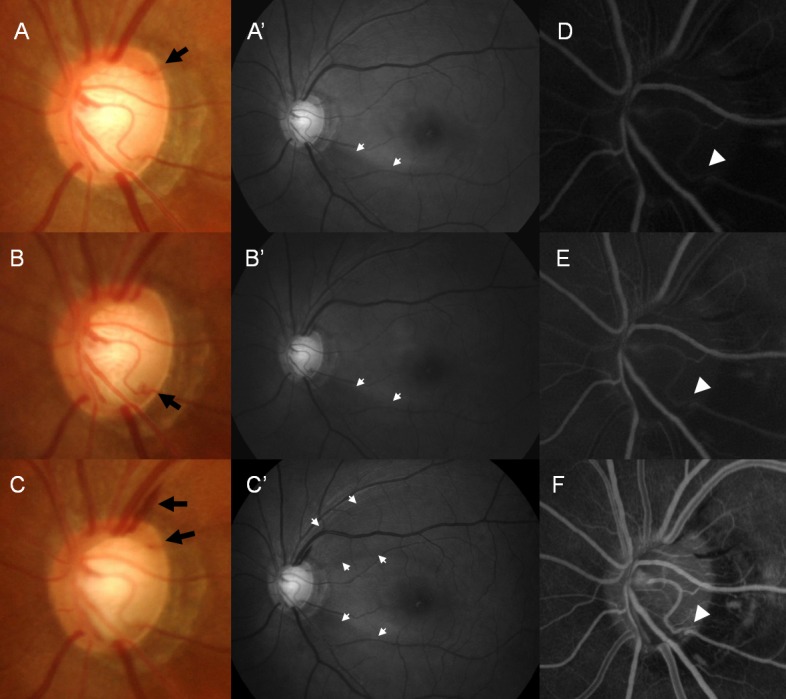
A 57-year-old male with normal-tension glaucoma. This patient had recurrent disc hemorrhages (DHs) at different locations of the optic disc in his left eye (A, B, and C, black arrow). DH occurred on the border of a localized retinal nerve fiber layer defect at the inferotemporal location (A’, B’, and C’, white arrow). Fluorescein angiography shows delayed arterial filling (white arrowhead) at the inferotemporal location in the early arteriovenous phase (D and E) and mid-arteriovenous phase (F). Although all the arteries are filled with fluorescein dye at the early arteriovenous phase (D and E), this artery at the inferotemporal location is not filled fully. This artery is kinked at the cup margin proximal to the location of DH.

The disc filling time, choroidal filling time, venous filling time, and frequency of disc leak were not different between eyes with DHs accompanying RNFL defects and eyes with DHs not related to RNFL defects (Table 3). However, arm-retina time was prolonged in eyes with DHs not related to RNFL defects (22.36 ± 5.66 seconds) compared to eyes with DHs related to RNFL defects (19.63 ± 3.63 seconds, *P* = 0.044). The arteriovenous transit time was prolonged in eyes with DH accompanying a RNFL defect (2.79 ± 1.72 seconds) compared to eyes with a DH not related to RNFL defect (1.79 ± 0.93 seconds, *P* = 0.029). The presence of a disc filling defect showed borderline significance between eyes with DHs accompanying RNFL defects (47.4%) and eyes with DHs not related to RNFL defects (18.8%, *P* = 0.077; Table 3). Representative cases are shown in Fig 1. Arteriovenous transit time is the time between the entrance of the opaque substance into the retinal artery (Fig 1, A-1; B-1; C-1) and entrance into the vein (Fig 1, A-2; B-2; C-2) from a distance of 2 optic discs. This was delayed to approximately 3 seconds in eyes with DH at the border of a localized RNFL defect (Fig 1, B’ and B”). Arm-retina time is the time from the administration of an opaque substance into the antecubital vein until it becomes visible in the retinal arteries (Fig 1, A-1; B-1; C-1). This was delayed to 23 seconds (Fig 1, C-1) in eyes with DH not related to a RNFL defect (Fig 1, C’ and C”) compared to normal controls (Fig 1, A-1, 15 seconds) and eyes with DH accompanying a RNFL defect (Fig 1, B-1, 19 seconds). At 29 seconds, almost all arteries and veins were filled with opaque substance in normal controls (Fig 1, A-3). However, just the arteries and a few veins were filled with opaque substance at around 27 and 30 seconds, respectively, in glaucomatous eyes with DH (Fig 1, B-3 and C-3).

**Table 3:** Comparison of parameters of fluorescein angiography between glaucoma eyes with disc hemorrhage (DH) occurring at the border of the localized retinal nerve fiber layer (RNFL) defect and DH occurring not related to RNFL defect.

	Accompanying RNFL defect	Not related to RNFL defect	*P* Value
Arm-retina time, second	19.63 ± 3.63	22.36 ± 5.66	0.044*
Arteriovenous transit time, second	2.79 ± 1.72	1.79 ± 0.93	0.029*
Disc filling time, second	10.15 ± 5.52	9.67 ± 3.91	0.155*
Choroidal filling time, second	11.03 ± 5.48	13.06 ± 7.77	0.393*
Venous filling time, second	13.29 ±5.01	14.42 ± 4.88	0.791*
Disc leak, n (%)	8 (42.1%)	4 (25.0%)	0.242^†^
Disc filling defect, n (%)	9 (47.4%)	3 (18.8%)	0.077^†^

Data are presented as mean ± standard deviation.

RNFL = retinal nerve fiber layer.

* Mann-Whitney U test.

^†^ Chi-square test.

**Fig 4:**
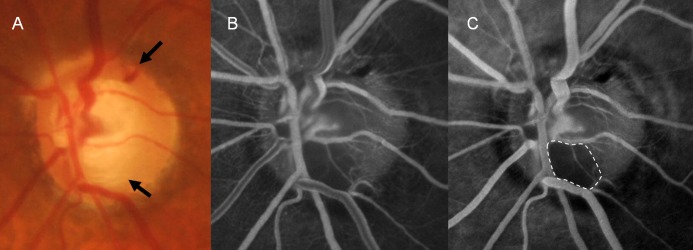
A 40-year-old female with primary open-angle glaucoma. This patient had recurrent disc hemorrhages (DHs) at the superotemporal and inferotemporal locations at the borders of localized retinal nerve fiber layer defects (A, black arrows). Disc filling defect is present on fluorescein angiography at the inferotemporal location (white dotted area), where a previous DH had occurred (B and C).

**Fig 5:**
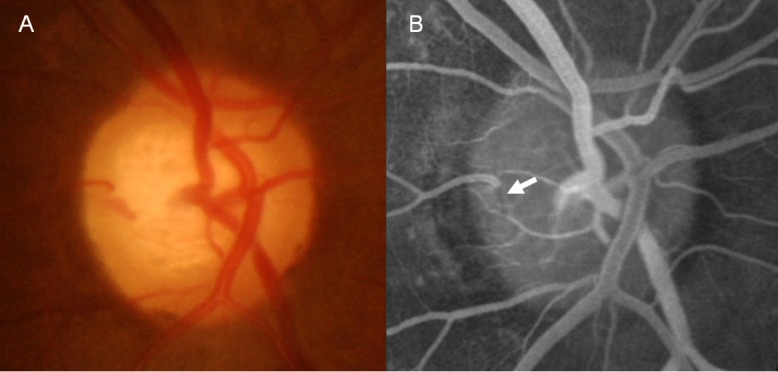
A 61-year-old female with normal-tension glaucoma. This patient had a disc hemorrhage (DH) at the 9 o’clock position at the cup base (white arrow). Fluorescein angiography revealed a thrombus, shown as a filling defect inside the vessel, where DH had occurred.

Among the 19 eyes with DH accompanying a RNFL defect, 11 (57.9%) had vessel filling defect or delayed filling, indicating blood flow stasis in these eyes. The location of vessel filling defects or delayed filling was the cup margin proximal to where the DH occurred. Any eyes with DH not related to a RNFL defect showed neither vessel filling defects nor delayed filling. One of the eyes with DH not related to a RNFL defect had a thrombus in the vessel at the location where the DH occurred. Representative cases are shown in Figs 2–5. Fig 2 shows the case of a 47-year-old female with NTG in her right eye. She had a localized RNFL defect in the inferotemporal region, and the VF showed superior nasal step defects at the corresponding region. DH occurred once during follow-up at the proximal border of the RNFL defect. Delay in venous filling proximal to the previous location of DH was observed from the mid-arteriovenous phase through the early late phase. At the late phase, all other veins around the ONH were filled with fluorescein dye except the vein proximal to the DH. This vein was kinked at the cup margin, and venous flow stasis was proximal to the cup margin where the DH occurred.

A 57-year-old male with NTG in his left eye had recurrent DH with an accompanying RNFL defect (Fig 3). The RNFL defect was located at the inferotemporal region at first, and a new RNFL defect appeared in the superotemporal region. Recurrent DHs occurred at the margins of the RNFL defects. Fluorescein angiography showed delayed arterial filling at the 5 o’clock position, where DH was previously present. Fluorescein filling was not present in this artery when venous filling had started at the early arteriovenous phase and also when all the other arteries were filled and the veins were halfway filled at the mid-arteriovenous phase. We could see the kink in the artery at the cup margin where the DH was present.

**Fig 6:**
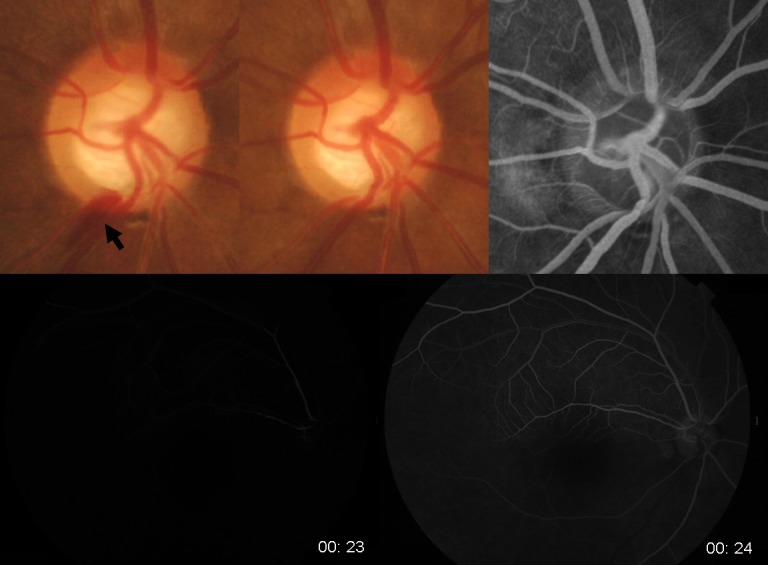
A 54-year-old female with normal-tension glaucoma. Disc hemorrhage not related to a localized retinal nerve fiber layer defect shows no specific findings suggesting hemodynamic changes, such as vessel filling defect or delayed filling, disc leak, and disc filling defects.

Another case with recurrent DH showed a disc filling defect at the inferior region of the optic disc (Fig 4). Superotemporal DH was present on fluorescein angiography, as well as a previous inferotemporal DH, both accompanying RNFL defects. A 61-year-old female case with NTG is shown in Fig 5. This eye had DH that was not related to a RNFL defect and that was located at the 9 o’clock position. In this case, we observed a thrombus at the location where the DH occurred. A case with DH not related to a RNFL defect had no apparent vessel filling defect or delayed filling, disc leak, and disc filling defects (Fig 6).

## Discussion

We observed that DHs occurring at the margins of RNFL defects had accompanying hemodynamic changes, which were not apparent in DHs not related to RNFL defects. By disc angiography, eyes with DHs at the margins of RNFL defects had prolonged arteriovenous transit time, which suggests blood flow stasis at the level of the artery to the vein. Approximately 47.4% had disc filling defects, showing blood flow stasis at the level of the capillary in the prelaminar region. Approximately 57.9% showed vessel filling defects or delayed filling at the cup margin in eyes with DHs occurring at the margins of RNFL defects. Cup margin was the most frequent proximal location of DH. Recurrent DH was significantly more frequent in eyes with DH occurring at the margins of a RNFL defect. This may show that DHs occurring at the margin of the cup or localized RNFL defect accompany blood flow stasis which may contribute to the development of DH and recurrence at that same site.

Virchow’s triad describes the three broad categories of factors that are thought to contribute to thrombosis: hemodynamic changes, hypercoagulability, and endothelial injury.[27] Hemodynamic changes are alterations in normal blood flow. These include blood flow stasis and turbulence. It has been speculated that passage of blood through the sclera to the vortex vein is oblique, and the IOP may exert a pressure on the vein walls.[2] This IOP-dependent resistance is the reason why DH occurs exclusively on the disc. We can also apply this concept to DHs occurring at the margins of RNFL defects. When the structure of the disc or RNFL changes as a result of the loss of the disc rim or RNFL due to glaucoma, the contours of the vessel will also change. Usually at the margin of the disc cup where there is a notch or at the margin of a localized RNFL defect, the course of the vessel may be acutely curved or kinked. These structural changes of the optic disc or RNFL may result in hemodynamic changes at that location, resulting blood flow stasis and formation of thrombosis that may contribute to subsequent DH.

This also explains why DH recurs in similar locations. Many reports show that most DH occurs within 2 clock hours of an area of neuroretinal rim notching, and recurred within 2 clock hours of the initial DH.[10, 21, 22, 28] Recurrent DH was frequent in eyes with a localized RNFL defect. This also shows that vessel changes due to glaucomatous structural changes are the cause of DH. One recent report showed DH was more frequent in eyes with enlargement of the RNFL defect and a RNFL defect enlarged in the direction of DH.[10] That study speculated that enlargement of RNFL defects cause loss of capillary network and that the border of a RNFL defect should be an active site for damaging the RNFL and surrounding capillary network. This means mechanical rupture of the capillaries results in DH when the RNFL defect is enlarged by glaucoma progression. For this to explain DH, recurrent DH would have to be present only in progressing glaucoma. However, whether recurrent DH is present in more progressively glaucomatous eyes is controversial.[21, 22]

Cases 2 and 3 were representative cases of vessel filling defect or delayed filling. A filling defect was presented proximal to the cup margin, where the vessel was reflected, in Case 2. A vessel showing delayed filling was kinked at the cup margin, where DH occurred, in Case 3. Both cases had DHs at the margins of localized RNFL defects and a proximal location of DH at the cup margin. In eyes with glaucomatous structural changes that further change the vessel contour, flow stasis and disturbance at that level may be the reason for DH. Both structural (glaucomatous disc and RNFL changes) and vascular aspects (blood flow stasis) contribute to the pathogenesis of DH. In particular, NTG has both structural and vascular aspects that may contribute to DH. NTG patients have risk factors for the vascular aspects. These include nocturnal dipping of blood pressure, ocular perfusion pressure fluctuation, orthostatic hypotension, heart-rate variability of autonomic dysfunction, and abnormal peripheral microcirculation.[29–33] NTG eyes have more localized RNFL defects compared to POAG eyes, and this structural aspect may explain why DH is more frequent in NTG.

Previous studies using fluorescein angiography in glaucoma show ischemic changes, persisting hypoperfusion, fluorescein leaks or filling defects of the optic disc.[23, 26, 34–36] It appears that reduced perfusion occurs early in the disease and accompaines capillary loss in the ONH.[23, 37, 38] Many investigators have applied fluorescein angiography to reveal the pathogenesis of glaucoma. All parameters stated in this study were prolonged in glaucoma compared to normal controls.[34, 35, 39, 40] Disc filling defects or leaks are found in around 30% of glaucomatous eyes, which is higher than that found in normal eyes.[25, 36] The most common reported finding from fluorescein angiography in glaucoma patients is delayed choroidal filling, especially peripapillary choroidal filling delay.[41, 42] No report has specifically investigated the changes in glaucoma with DH. Spaeth et al. described some cases of DH, but did not find a difference between glaucoma with and without DH.[23] In our study, we did not include any patients who had glaucoma without DH. Our values of the parameters of fluorescein angiography and the frequency of disc leaks or filling defects seem to be similar to previous studies. However, the distinct characteristic of glaucoma patients with DH was prolonged arm-retina time. This time was significantly prolonged in glaucomatous eyes with DHs not related to RNFL defects. Further investigation to confirm this finding may be needed.

Our study has several limitations that must be acknowledged. First, the sample size was small and there are possibilities of selection bias according to the location of DH. A second limitation is that it is difficult to generalize our findings since only Korean individuals were included. Also, our glaucoma patients had early damage (mean MD of approximately -3.55 dB). The number of DHs may have been underestimated in some patients; we could only use the number of detected DHs. However, this is why we included patients who were followed-up at least 5 years with photographs taken every 6–12 months. By 5 years, the incidence of DH has typically plateaued, with very few initial DHs found after this time.[19] Finally, this was a cross-sectional study and angiography was performed 3 months after the presence of DH. Wether our findings from angiography may contribute to the occurrence of DH or it was just a detected phenomenon may need further investigations.

In conclusion, we found that hemodynamic characteristics were different between eyes with DH accompanying glaucomatous structural changes of the optic disc or RNFL compared to eyes with DH not related to these structures. Eyes with DH related to glaucomatous structural changes of the optic disc or RNFL had delay in arteriovenous transit time, apparent vessel filling defects or delayed filling, and more frequent disc filling defects. These eyes showed more frequent recurring DH, and the location of DH was mainly at the cup margin, where the vessel contour changes due to the structure of the optic disc or RNFL. Together, these results show that vascular and hemodynamic changes due to glaucomatous structural changes may contribute to the development of DH.
